# Nocturnal CPAP improves walking capacity in COPD patients with obstructive sleep apnoea

**DOI:** 10.1186/1465-9921-14-66

**Published:** 2013-06-19

**Authors:** Tsai-Yu Wang, Yu-Lun Lo, Kang-Yun Lee, Wen-Te Liu, Shu-Min Lin, Ting-Yu Lin, Yung-Lun Ni, Chao-Yung Wang, Shu-Chuan Ho, Han-Pin Kuo

**Affiliations:** 1 Department of Thoracic Medicine, Chang Gung Memorial Hospital and Chang Gung University, School of Medicine, Taipei, Taiwan; 2 Division of Pulmonary, Department of Internal Medicine, Shuang Ho Hospital, Taipei, Taiwan; 3 School of Respiratory Therapy, College of Medicine, Taipei Medical University, Taipei, Taiwan; 4 Department of Chest Medicine, Buddhist Tzu Chi General Hospital, Taichung Branch, Taichung, Taiwan; 5 Department of Cardiology Medicine, Chang Gung Memorial Hospital and Chang Gung University, School of Medicine, Taipei, Taiwan

**Keywords:** Chronic obstructive pulmonary disease, Obstructive sleep apnoea, Walking capacity, Autonomic dysfunction, Continuous positive airway pressure

## Abstract

**Background:**

Exercise limitation is an important issue in patients with chronic obstructive pulmonary disease (COPD), and it often co-exists with obstructive sleep apnoea (overlap syndrome). This study examined the effects of nocturnal continuous positive airway pressure (CPAP) treatment on walking capacity in COPD patients with or without obstructive sleep apnoea.

**Methods:**

Forty-four stable moderate-to-severe COPD patients were recruited and completed this study. They all underwent polysomnography, CPAP titration, accommodation, and treatment with adequate pressure. The incremental shuttle walking test was used to measure walking capacity at baseline and after two nights of CPAP treatment. Urinary catecholamine and heart rate variability were measured before and after CPAP treatment.

**Results:**

After two nights of CPAP treatment, the apnoea-hypopnoea index and oxygen desaturation index significantly improved in both overlap syndrome and COPD patients, however these changes were significantly greater in the overlap syndrome than in the COPD group. Sleep architecture and autonomic dysfunction significantly improved in the overlap syndrome group but not in the COPD group. CPAP treatment was associated with an increased walking capacity from baseline from 226.4 ± 95.3 m to 288.6 ± 94.6 m (P < 0.05), and decreased urinary catecholamine levels, pre-exercise heart rate, oxygenation, and Borg scale in the overlap syndrome group. An improvement in the apnoea-hypopnoea index was an independent factor associated with the increase in walking distance (r = 0.564).

**Conclusion:**

Nocturnal CPAP may improve walking capacity in COPD patients with overlap syndrome.

**Trial registration:**

NCT00914264

## Introduction

Mortality due to chronic obstructive pulmonary disease (COPD) is increasing, and COPD is a serious health burden worldwide. The characteristic physiological impairments are airflow limitation, air-trapping, hyper-inflation, dyspnoea, and exercise limitation [1]. Patients with COPD and exercise limitation will adopt a more sedentary lifestyle and give up the more strenuous physical activities [2,3], which eventually leads to de-conditioning and ultimately further exercise limitation [4]. Moreover, exercise limitation is associated with morbidity and mortality [5,6]. As such, finding ways to break this vicious cycle of exercise limitation is an important issue in COPD. Exercise limitation is influenced by age, body weight, dyspnoea sensation, pulmonary function, skeletal muscles, hypoxemia, cardio-vascular function, autonomic dysfunction, and emotion [7]. Nocturnal intermittent hypoxemia and sleep fragmentation have also been shown to be associated with impaired walking performance in older men [8]. In COPD patients, it has been suggested that hypoxemia during exercise limits walking performance, but that this is improved by oxygen supply during exercise [9,10]. However, whether nocturnal intermittent hypoxemia affects walking performance remains unclear.

Obstructive sleep apnoea (OSA) is characterized by recurrent pharyngeal collapse with intermittent hypoxemia during sleep and subsequent repetitive arousal to maintain ventilation. Concomitant COPD and OSA, termed the overlap syndrome, is not rare and affects at least 1% of the general population [11-13]. Nocturnal continuous positive airway pressure (CPAP) via a pneumatic splint is effective in maintaining upper airway patency, and is standard treatment for OSA [14]. In addition, it can reverse sleep fragmentation, nocturnal intermittent hypoxemia and autonomic dysfunction. Therefore, it is reasonable to assume that reversing intermittent hypoxemia, sleep fragmentation and autonomic dysfunction will improve walking performance in overlap syndrome patients. The aim of this study was to clarify whether walking performance significantly increases in COPD patients with OSA after nocturnal CPAP treatment.

## Materials and methods

This prospective, controlled, observational study was performed at one sleep centre of a tertiary hospital (Chang Gung Memorial Hospital) from January 2009 to May 2012. The local Ethics Committee of Chang Gung Memorial Hospital approved the research protocol (NCT00914264) and each patient provided written informed consent.

### Subjects

Outpatients with stable COPD were enrolled from the thorax department of Chang Gung Memorial Hospital. The inclusion criteria were FEV_1_/FVC less than 70%, a smoking history of at least 10 pack-years, and snoring (≥ 3 nights/week). The exclusion criteria were an age younger than 40 years, bronchiectasis, acute exacerbation within 2 months of the study, renal insufficiency, heart failure, neuromuscular disease, mood disorder, malignancy, claustrophobia, chronic respiratory failure (PaO_2_ < 60 mmHg or PaCO_2_ > 50 mmHg in room air), or sleep disorders other than OSA.

### Study design

This study investigated the effects of two nights of CPAP treatment on the walking capacity of COPD patients with or without OSA. All patients underwent polysomnography on the first night to identify those with OSA. The incremental shuttle walking test (ISWT) was then performed by all of the study patients on the morning of the second day. The patients also underwent CPAP accommodation and titration according to published guidelines to determine the optimal pressure on the second night [15]. On the third night, the optimal pressure was applied to all of the study patients. The ISWT was performed again on the morning of the fourth day. Urine was collected from 10 pm to 7 am on the first and third nights and sent to the central lab for catecholamine analysis. The assistants and technicians who helped with the ISWT and urine analysis were blinded to the severity of OSA. In addition, the patients were not aware of the severity of OSA. All patients received regular rehabilitation programs, and medical care did not change during the study period.

### Polysomnography and CPAP titration

Polysomnography (Alice 5, Respironics) was performed on all patients using standard techniques on the first night. Sleep stages and arousals were scored according to the AASM criteria [16]. Respiratory efforts were measured by respiratory plethysmography, and arterial oxygen saturation was measured by pulse oximetry. Established criteria were used to score respiratory events such as hypopnoea, obstructive apnoea, central apnoea, mixed type apnoea, and Cheyne-Stokes respiration [17]. Apnoea was defined as oronasal flow cessation for more than 10 seconds. Hypopnoea was defined as a 50% reduction in oronasal flow for more than 10 seconds or a 30% reduction followed by arousal or more than 3% decrease in SaO2. Based on the polysomnography results, OSA was defined as an apnoea/hypopnoea index (AHI) > 15 per hour, of which ≥ 50% were obstructive. CPAP titration to determine optimal pressure was performed according to standard guidelines [15].

### Heart rate variability and data analysis

After waking, all patients were requested to breathe regularly and as smoothly as possible in a quiet environment. CPAP was removed as soon as the patients awoke in the morning. The first available 5-minute RR data after waking without CPAP was identified for each patient on the second and fourth mornings. The details of the measurement of heart rate variability are described in the online supplement.

### Urinary catecholamine levels

Patients were requested to collect urine samples from 10:00 pm to 7:00 am. The urine samples were collected in polyethylene containers, acidified with 6 M HCl as a preservative, and stored at 2-8°C until analysis [18,19]. Urinary levels of catecholamines were measured by high performance liquid chromatography with a Bio-Rad kit at the central laboratory of our hospital.

### Incremental shuttle walking test

The ISWT was used to determine maximal walking capacity [20,21]. All of the patients performed the ISWT at least once before the study. The test was then repeated on the mornings of the second (baseline) and fourth (after nocturnal CPAP treatment) days. The details of the ISWT are described in the online supplement and a previous study [20].

### Statistical analysis

Data were expressed as group percentages (categorical variables) or mean ± standard deviation (SD, continuous variables), and compared between pre- and post-CPAP treatment. Categorical variables were compared by the chi-square or Fisher’s exact test where appropriate. The paired t-test was used to compare continuous variables with normal distribution and paired samples. The Wilcoxon matched signed rank test was used for paired samples not distributed normally (all P < 0.05). The Pearson product correlation coefficient was used to examine correlations between variables and the change in walking distance. Multivariate linear regression analysis was used to determine independent factors affecting the change in walking distance. All analyses were performed using the SPSS software package version 13.0 and Prism 5, and a P value of less than 0.05 was considered to indicate statistical significance.

## Results

### Subject characteristics and polysomnography results

**Figure 1:**
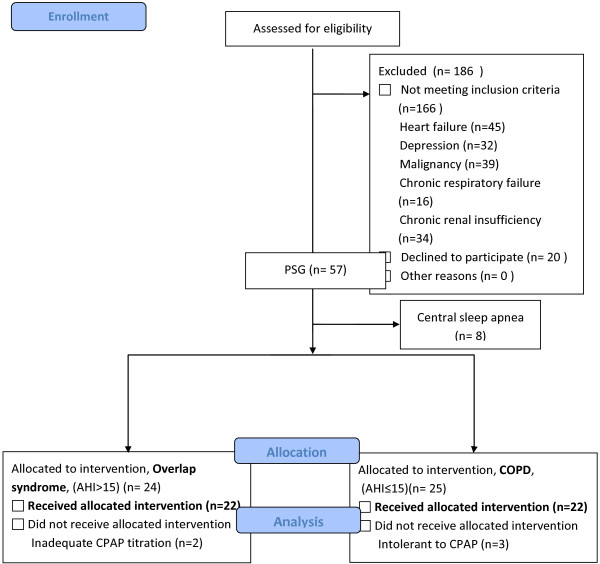
Flow chart of the study design.

A flow chart of the study design is shown in Figure 1, and the patients’ characteristics at baseline are shown in Table 1. Forty-four COPD patients were recruited and completed this study. There was a higher percentage of overlap syndrome patients (50%) in this study than in previous reports, which may be explained by the inclusion criterion of habitual snoring [22]. There were no significant differences in age, gender, body mass index, pulmonary function, arterial blood gas, medications and comorbidities between the groups, however the overlap syndrome patients seemed to be sleepier and have poorer sleep quality than the COPD patients. The mean body mass index in the overlap syndrome group was 24.9 ± 3.5 kg/m^2^, which is much lower than that reported in Caucasians, but compatible with Asian OSA patients [23]. There were no significant differences in baseline total sleep time, sleep efficiency, mean oxygen saturation during sleep or sleep architecture between the groups (Table 2). The mean AHI in the overlap syndrome group was 39.2 ± 16.5/h of sleep, indicating that most of the patients had severe OSA (Table 2). CPAP treatment did not affect sleep efficiency or total sleep time in either group, however it significantly decreased AHI and oxygen desaturation index (ODI) in both groups. This treatment effect was significantly greater in the overlap syndrome group than in the COPD group. CPAP treatment also significantly increased the average and minimal SaO_2_ in both groups (Table 2). CPAP treatment improved sleep architecture in the patients with overlap syndrome, but not in those with COPD, by decreasing the proportion of stage 1 sleep (from baseline 23.0 ± 14.0% to 15.4 ± 8.9%, P < 0.05) and increasing slow wave sleep (from baseline 2.3 ± 3.9% to 5.2 ± 6.1%, P < 0.05) as well as REM stage sleep (from baseline 8.5 ± 7.1% to 16.4 ± 10.3%, P < 0.05) (Table 2).

**Table 1:** Patient characteristics

	**Overlap syndrome n = 22**	**COPD n = 22**	***p-*value**
Age	70.0 ± 8.3	71.7 ± 8.5	0.495
Male, n (%)	21 (96.7)	22 (100)	0.999
BMI	24.9 ± 3.5	22.9 ± 3.0	0.054
Smoking, pack-years	52.3 ± 21.5	50.8 ± 26.4	0.837
ESS	12.7 ± 4.5	8.3 ± 3.5	0.004
PSQ	11.8 ± 4.0	7.5 ± 3.7	0.001
Emphysema predominant, n (%)	5 (22.7)	14 (63.6)	0.014
**Pulmonary function test**			
FEV_1_/FVC	57.5 ± 12.7	54.5 ± 9.6	0.197
FEV_1_ (L)	1.09 ± 0.51	0.95 ± 0.45	0.227
FEV_1_ (% predicted)	52.7 ± 28.6	45.1 ± 19.2	0.431
FVC (L)	1.87 ± 0.69	1.70 ± 0.60	0.348
FVC (% predicted)	63.3 ± 25.1	57.2 ± 21.7	0.378
**Arterial blood gas**			
pH	7.4 ± 0.0	7.4 ± 0.0	0.672
PaCO_2_ (mm Hg)	41.8 ± 3.8	40.6 ± 3.8	0.354
PaO_2_ (mmHg)	77.5 ± 11.9	79.2 ± 9.0	0.581
**Medication**			
LABA + ICS, n (%)	20 (90.9)	19 (86.4)	0.565
LAMA, n (%)	18 (81.8)	17 (77.3)	0.907
Theophyllines, n (%)	6 (27.3)	2 (9.1)	0.375
**Comorbidities**			
DM, n (%)	0 (0)	1 (4.5)	-
HTN, n (%)	10 (45.4)	8 (36.4)	0.348
CAD, n (%)	1 (4.5)	1 (4.5)	0.823
PAOD, n (%)	0 (0)	0 (0)	-

Data are presented as mean ± SD, or number (percentage).

*BMI* body mass index, *ESS* Epworth sleepiness scale, *PSQ* Pittsburg sleep quality, *FEV1* forced expiratory volume in one second, *FVC* forced volume capacity, *ICS* inhaled corticosteroids, *LABA* long-acting β_2_ agonist, *LAMA* long-acting muscarinic antagonist, *DM* diabetes mellitus, *HTN* hypertension, *CAD* coronary artery disease, *PAOD* peripheral arterial occlusion disease.

**Table 2:** Polysomnographic report: patients with and without CPAP treatment

	**Overlap syndrome**			**COPD**			
	**n = 22**			**n = 22**			
	**Baseline**	**With CPAP**	**P**	**Baseline**	**With CPAP**	**P**	^†^**P**
Total sleep time (minutes)	294.3 ± 80.5	290.1 ± 94.9	0.758	287.8 ± 77.9	273.2 ± 71.7	0.408	0.760
Sleep efficiency (%)	67.2 ± 12.8	68.6 ± 14.3	0.833	66.3 ± 14.2	65.2 ± 12.9	0.650	0.699
AHI event. h^-1^	39.2 ± 16.5	4.6 ± 2.9	0.001	7.5 ± 4.1*	1.6 ± 1.4	0.001	0.001
ODI event. h^-1^	20.4 ± 16.2	3.1 ± 2.9	0.001	3.1 ± 2.2*	0.9 ± 1.1	0.001	0.001
Average SaO_2_%	93.3 ± 2.2	94.5 ± 1.7	0.011	93.8 ± 2.0	94.9 ± 2.1	0.001	0.866
Lowest SaO_2_%	80.3 ± 8.8	89.3 ± 3.9	0.001	87.0 ± 4.6*	90.2 ± 4.3	0.005	0.089
Wake%	26.6 ± 10.3	21.8 ± 13.7	0.211	25.5 ± 14.2	26.9 ± 12.7	0.578	0.124
N1%	23.0 ± 14.0	15.4 ± 8.9	0.010	20.3 ± 14.1	16.5 ± 8.7	0.079	0.296
N2%	39.5 ± 12.6	41.2 ± 14.9	0.700	34.0 ± 11.8	33.2 ± 11.6	0.941	0.751
N3%	2.3 ± 3.9	5.2 ± 6.1	0.001	7.5 ± 9.4	9.2 ± 11.4	0.235	0.191
REM%	8.5 ± 7.1	16.4 ± 10.3	0.004	12.2 ± 7.7	13.9 ± 7.5	0.144	0.026
**CPAP pressure** (cm H_2_O)		6.8 ± 1.8			4.9 ± 0.9*		

*P < 0.05; when compared with baseline overlap syndrome group.

P values represent comparisons between baseline and with CPAP in each corresponding group.

^†^P values represent comparisons with regards to the changes with CPAP treatment between overlap syndrome and COPD in each corresponding group.

*AHI* apnoea/hypopnea index, *ODI* oxygen desaturation index, *SWS* slow wave sleep, *REM* rapid eye movement, *CPAP* continuous positive airway pressure.

### Heart rate variability and urinary catecholamine levels

Four patients (18.2%) in the overlap syndrome group and two patients (9.1%) in the COPD group were excluded from heart rate variability analysis due to arrhythmias. The patients with overlap syndrome (n = 18) had significantly higher baseline LF (46.6 ± 16.1 nu, P < 0.05), LF/HF (1.8 ± 1.2, P < 0.05) and lower HF (32.2 ± 12.0 nu, P < 0.05) on awakening from sleep than the patients with COPD (31.8 ± 10.8 nu, 0.9 ± 0.6 and 41.9 ± 12.1 nu, respectively, n = 20) (Table 3). To further investigate the decrease in sympathetic activity after CPAP treatment in the overlap syndrome patients, urine catecholamine measurements were added into the protocol of this study. Urine samples were collected from all of the study patients except for four patients with overlap syndrome who refused the urine study. The results revealed higher levels of norepinephrine and epinephrine in the patients with overlap syndrome (17.0 ± 8.4 μg and 2.4 ± 0.9 μg, respectively, n = 12) than those in the COPD patients (11.1 ± 5.3 μg and 1.5 ± 0.6 μg, respectively, P < 0.05, n = 12) (Table 3). After CPAP treatment, LF (31.3 ± 16.7 nu, P < 0.05, n = 18), LF/HF (0.9 ± 0.7, P < 0.05, n = 18), and urinary levels of norepinephrine and epinephrine (10.4 ± 6.4 μg and 1.4 ± 0.7 μg, respectively, P < 0.05, n = 12) significantly decreased in the overlap syndrome group, but not in the COPD group. HF also significantly increased after CPAP treatment in the overlap syndrome group (43.1 ± 19.1 nu, P < 0.05, n = 18), but not in the COPD group (Table 3).

**Table 3:** Heart rate variability and urine catecholamine: patients with and without CPAP treatment

**Heart rate variability**	**Overlap syndrome n = 18**^**#**^			**COPD**		
				**n = 20**^**@**^		
	**Baseline**	**With CPAP**	**p**	**Baseline**	**With CPAP**	**p**
LF (nu)	46.6 ± 16.1	31.3 ± 16.7	0.001	31.8 ± 10.8*	28.7 ± 20.4	0.401
HF (nu)	32.2 ± 12.0	43.1 ± 19.1	0.029	41.9 ± 12.1*	43.6 ± 19.3	0.287
LF/HF	1.8 ± 1.2	0.9 ± 0.7	0.003	0.9 ± 0.6*	1.2 ± 1.9	0.514
**Urine catecholamines**	**Overlap syndrome n = 12**^**$**^			**COPD**		
				**n = 12**^**$**^		
	**Baseline**	**With CPAP**		**Baseline**	**With CPAP**	
Norepinephrine, ug	17.0 ± 8.4	10.4 ± 6.4	0.003	11.1 ± 5.3*	11.9 ± 7.5	0.485
Epinephrine, ug	2.4 ± 0.9	1.4 ± 0.7	0.025	1.5 ± 0.6*	1.8 ± 0.8	0.374

*P < 0.05; when compared with baseline overlap syndrome group.

P values represent comparisons between before and after CPAP in each corresponding group.

*LF* low frequency, *HF* high frequency *nu* normalized unit.

### Incremental shuttle walking test

There were no significant differences in baseline walking distance, pre-exercise or post-exercise Borg scale, oxygen saturation, heart rate or inspiratory capacity (IC) between the overlap syndrome and COPD groups (Table 4). For patients with overlap syndrome, CPAP treatment significantly increased walking distance (from baseline from 226.4 ± 95.3 m to 288.6 ± 94.6 m, P < 0.05), and pre-exercise oxygenation (from baseline 94.5 ± 2.2% to 95.5 ± 1.9%, P < 0.05), and significantly decreased pre-exercise Borg scale (from 1.5 ± 1.5 to 0.8 ± 1.1, P < 0.05) and heart rate (from 94.3 ± 17.7/min to 87.3 ± 15.8/min, P < 0.05) (Table 4). CPAP treatment did not significantly affect pre-exercise IC or delta-IC in the patients with overlap syndrome (Table 4). In the patients with COPD, CPAP treatment also improved walking distance, however, the increase in walking distance (delta-distance) in the overlap syndrome group (62.3 ± 24.9 m) was significantly greater than that in the COPD group (15.1 ± 33.6 m). In addition, CPAP treatment did not improve pre-exercise Borg scale, heart rate, or IC in the COPD group (Table 4).

**Table 4:** Incremental shuttle walking test: before and after CPAP treatment

**Variable**	**Overlap syndrome**			**COPD**			
	**n = 22**			**n = 22**			
	**Before**	**After**	**p**	**Before**	**After**	**p**	^†^**P**
		**CPAP**		**CPAP**	**CPAP**			**CPAP**
Distance (metre)	226.4 ± 95.3	288.6 ± 94.6	0.001	257.5 ± 89.1	272.5 ± 98.4	0.035	0.001
Borg scale (pre-exercise)	1.5 ± 1.5	0.8 ± 1.1	0.040	1.4 ± 1.3	0.7 ± 1.1	0.064	0.832
Borg scale (post-exercise)	4.9 ± 0.9	4.8 ± 1.1	0.851	5.0 ± 1.1	4.7 ± 1.2	0.450	0.596
SaO_2_ (pre-exercise)	94.5 ± 2.2	95.5 ± 1.9	0.014	94.6 ± 2.7	95.3 ± 2.2	0.046	0.351
SaO_2_ (post-exercise)	88.8 ± 4.4	87.8 ± 6.3	0.827	85.1 ± 6.0	84.6 ± 5.7	0.717	0.972
Heart rate (pre-exercise)	94.3 ± 17.7	87.3 ± 15.8	0.009	93.2 ± 15.6	93.4 ± 16.1	0.999	0.026
Heart rate (post-exercise)	136.3 ± 24.2	125.0 ± 22.8	0.018	125.3 ± 29.3	134.8 ± 29.6	0.135	0.010
Delta-IC (L)	−0.04 ± 0.23	−0.08 ± 0.17	0.489	−0.13 ± 0.21	−0.12 ± 0.24	0.979	0.548
IC pre-exercise (L)	1.37 ± 0.48	1.44 ± 0.48	0.184	1.34 ± 0.42	1.32 ± 0.42	0.687	0.171
IC post-exercise (L)	1.33 ± 0.48	1.36 ± 0.46	0.586	1.21 ± 0.42	1.20 ± 0.49	0.672	0.225

P values represent comparisons between before and after CPAP in each corresponding group.

^†^P values represent comparisons with regards to the changes with CPAP treatment between overlap syndrome and pure COPD in each corresponding group.

*IC* inspiratory capacity.

**Figure 2:**
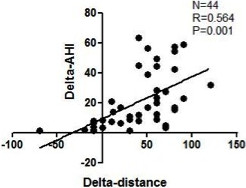
Correlation between the changes in apnoea-hypopnoea index (delta-AHI) and the changes in walking distance (delta-distance) with CPAP treatment in the patients with overlap syndrome.

Overall, the increase in walking distance (delta-distance) after CPAP treatment was significantly related to the attenuation of sleep apnoea/hypopnoea episodes (delta-AHI) (r = 0.564, P = 0.001) (Figure 2) and de-saturation episodes (delta-ODI) (r = 0.402, P = 0.007). In univariate analysis, only delta-AHI and delta-ODI were significantly correlated with delta-distance (Table E1). Although the sleep architectures (SWS and REM) were significant changed after CPAP treatment, there were no significant correlations between delta-distance and delta-REM as well as delta-distance and delta-SWS no matter they were represented in minutes or % of TST. Multivariate analysis revealed that delta-AHI was the only independent factor associated with delta-distance.

In the patients with overlap syndrome, 14 (63.6%) received continuous CPAP treatment for three months. Of these patients, 10 patients had adequate CPAP compliance, which was defined as the use of CPAP > 4 hours per night and > 5 days per week. Eight (36.4%) patients refused continuous CPAP treatment. Three of these eight patients refused CPAP treatment because of the personal issues and the other five patients did not feel much better after two nights of CPAP treatment. The AHI after three months of follow up was not significantly different between the continuous CPAP and non-CPAP treatment groups (36.2 ± 19.1, n = 10 and 35.6 ± 14.2, n = 8, respectively). In addition, there was no significant change in pulmonary function in terms of FEV1 in either the continuous CPAP treatment group (53.9 ± 30.9% predicted, n = 10) or non-CPAP treatment group (47.4 ± 23.9% predicted, n = 8) when compared to the baseline values before treatment (52.7 ± 31.3% predicted, n = 10 and 46.1 ± 24.5% predicted, n = 8, respectively). The continuous CPAP treatment group maintained the increase in walking distance with CPAP treatment after three months of follow-up (57.0 ± 39.2 M) (Online supplement Figure 1). However, the walking distance returned to baseline in non-CPAP treatment group after three months of follow-up.

## Discussion

This study demonstrated that two nights of nocturnal CPAP treatment was associated with an improvement in walking capacity in COPD patients with OSA. In addition, the increase in walking distance was clinically significant and relevant [24]. To date, this is the first study to report the impact of CPAP treatment on COPD patients with OSA.

Exercise performance is an important issue for COPD patients because it is associated with mortality, hospitalization, and acute exacerbation [2-6]. Poor exercise performance also leads to physical inactivity, and subsequently to muscle atrophy and de-conditioning, which causes a further decrease in exercise performance leading to a vicious cycle [4]. Many factors are associated with exercise performance including age, body weight, pulmonary function, oxygenation, dyspnoea sensation, skeletal muscles, cardiovascular disease, cognitive function, and obstructive sleep apnoea [7,8,25]. Patients with heart failure, renal insufficiency, neuromuscular disease, mood or sleep disorders other than OSA, and chronic respiratory failure were excluded from this study so as to focus on the effects of OSA on the walking capacity of COPD patients.

Hypoxemia, one of the cardinal features of OSA, is known to be more severe in overlap syndrome than in either individual syndrome, and this was also found in the current study. In an animal study, chronic intermittent hypoxemia was found to increase diaphragmatic and limb muscle fatigue in rats [26]. In humans, inspiratory muscle dysfunction has been noted in severe obstructive sleep apnoea [27]. This suggests that intermittent hypoxemia impairs muscle function including accessory muscles, diaphragm and limb muscles. Moreover, skeletal muscle atrophy and dysfunction, a common complication in COPD, contribute to increased nocturnal hypoxemia. The use of nocturnal CPAP to break up this vicious cycle may act by attenuating skeletal muscle dysfunction, which may in turn improve the walking capacity in patients with overlap syndrome.

Sleep deprivation has been documented to be associated with poor exercise performance [28]. In addition, poor exercise performance has also been reported in patients with OSA, which is characterised by sleep fragmentation, worsened sleep architecture, and subjective daytime sleepiness [29]. Similar results were also demonstrated in this study; however, there was no significant association between the increase in walking distance (delta-distance) after CPAP treatment and the improvement in sleep architecture. The existence of ß-errors is possible, and further large-scale studies are needed to validate our findings.

Autonomic dysfunction, another cardinal feature of OSA, can be reversed by CPAP, and is also noted during peak exercise in COPD patients [30,31]. In this study, we found that the patients with overlap syndrome had higher LF, LF/HF ratio, urine norepinephrine and epinephrine levels compared to the COPD patients, suggesting that a higher sympathetic activity exists in patients with overlap syndrome. This autonomic dysfunction has a negative impact on the walking performance of COPD patients with OSA. Increased sympathetic drive leads to inadequate blood flow, local inflammation, and abnormal skeletal muscle metabolism, which worsen the walking capacity of COPD patients [32]. The autonomic dysfunction and urine catecholamine levels in the patients with overlap syndrome after CPAP treatment decreased to the same values as the baseline of the COPD patients, indicating that CPAP reversed the autonomic dysfunction mostly caused by OSA. The significant decrease in pre-exercise heart rate after CPAP treatment in the patients with overlap syndrome may also represent an improvement in autonomic dysfunction after CPAP treatment. However, the extent to which this autonomic dysfunction affected the exercise capacity of the overlap syndrome patients is unknown.

CPAP treatment has been reported to improve survival in patients with overlap syndrome [33], and in this study we found that CPAP treatment improved the walking capacity in the patients with overlap syndrome. Whether this improvement in exercise capacity can be translated into an improvement in the mortality rate as reported previously [5,6,34] warrants a prospective, multi-center study with a larger study population and a longer observation time.

For the issues about the imbalance of emphysema component between groups are needed to be further discussion. All patients enrolled into this study were arranged HRCT. The severity of emphysema was assessed by an independent radiologist who was blinded to this study according to the Goddard scoring system [35]. In addition, the percentage of emphysema predominant type in overlap syndrome group is 5/22 (22.7%) and that in COPD group is 63.6% (14/22) (P = 0.014). Therefore, the result of this study should be caution to apply to emphysema predominant type COPD with OSA. In emphysema predominant type COPD, the lung volume increases, which decreases the severity of obstructive sleep apnea as Heinzer RC et al. described [36]. In addition, snores is one of our inclusion criteria that maybe the reason why the percentage of emphysema predominant type is relative low in overlap syndrome group.

In conclusion, the presence of OSA in COPD patients affects sleep architecture, increases hypoxemia episodes during sleep and increases sympathetic drive on awakening that together worsen the exercise capacity. Nocturnal CPAP treatment attenuated OSA-related dysfunction and then significantly increased the exercise capacity in the patients with overlap syndrome. However, this study is an observational study with a small sample size. Therefore, a large-scale double-blinded randomised control trial is needed to draw a more definite conclusion.

## Competing interests

The authors declare that they have no competing interests.

## Authors’ contributions

TYW: contributed to conceptualization and design of this study; collection, analysis, and interpretation of the data; and preparation of the manuscript. YLL: contributed to conceptualization and design of this study; collection, analysis, and interpretation of the data; and preparation of the manuscript. KYL, WTL, SML, TYL, YLN, SCH: contributed to collection, analysis, and interpretation of the data and preparation of the manuscript. CYW: performed cardiac echography to determine the ejection fraction of each patient. HPK: contributed to conceptualization and design of the study; collection and interpretation of the data; and preparation of the manuscript. All authors read and approved the final manuscript.
